# Follicular dendritic cells, conduits, lymphatic vessels, and high endothelial venules in tertiary lymphoid organs: Parallels with lymph node stroma

**DOI:** 10.3389/fimmu.2012.00350

**Published:** 2012-11-30

**Authors:** Sharon Stranford, Nancy H. Ruddle

**Affiliations:** ^1^Department of Biological Sciences, Mount Holyoke CollegeSouth Hadley, MA, USA; ^2^Department of Epidemiology of Microbial Diseases, Yale School of Public HealthNew Haven, CT, USA; ^3^Department of Immunobiology, Yale University School of MedicineNew Haven, CT, USA

**Keywords:** autoimmunity, chronic inflammation, cancer, secondary lymphoid organ, tertiary lymphoid tissue

## Abstract

In this communication, the contribution of stromal, or non-hematopoietic, cells to the structure and function of lymph nodes (LNs), as canonical secondary lymphoid organs (SLOs), is compared to that of tertiary lymphoid tissue or organs (TLOs), also known as ectopic lymphoid tissues. TLOs can arise in non-lymphoid organs during chronic inflammation, as a result of autoimmune responses, graft rejection, atherosclerosis, microbial infection, and cancer. The stromal components found in SLOs including follicular dendritic cells, fibroblast reticular cells, lymphatic vessels, and high endothelial venules and possibly conduits are present in TLOs; their molecular regulation mimics that of LNs. Advances in visualization techniques and the development of transgenic mice that permit *in vivo* real time imaging of these structures will facilitate elucidation of their precise functions in the context of chronic inflammation. A clearer understanding of the inflammatory signals that drive non-lymphoid stromal cells to reorganize into TLO should allow the design of therapeutic interventions to impede the progression of autoimmune activity, or alternatively, to enhance anti-tumor responses.

## INTRODUCTION

The non-hematopoietic or stromal cells present in secondary lymphoid organs (SLOs) – lymph nodes (LNs), spleen, Peyer’s patches (PP)- provide the structural and functional underpinnings that allow the most efficient encounter of lymphocytes with their cognate antigens. In this communication, we compare and analyze the contribution of stromal cells in SLOs to a particular type of cellular accumulation, the tertiary lymphoid tissue/organ (TLO).

The immune system generates a repertoire of antigen specific lymphocytes during development of these cells in primary lymphoid organs (the thymus, fetal liver, and bone marrow). Mature lymphocytes regularly transit through SLOs where encounter with cognate antigen is facilitated, leading to an effective immune response. Strategically positioned throughout the body, LNs are encapsulated, highly organized structures; T cells and dendritic cells (DCs) are concentrated in the paracortical region, with B cells and follicular DCs (FDCs) in the cortical region. In the mature LN, resident stromal cells play several critical roles, providing: (1) a meshwork supporting lymphocyte attachment, (2) chemokine signals directing cell subset positioning, (3) a gateway for entrance for lymphocytes (high endothelial venules; HEVs), (4) connections between nodes (lymphatic vessels; LVs), (5) entry points for antigen and antigen presenting cells (APCs; afferent LVs), (6) microvessels, allowing rapid import of small antigens and chemical signals (conduits formed by fibroblastic reticular cells; FRCs), and (7) sites of self-antigen presentation (FRCs, eTACs, LVs).

**FIGURE 1:**
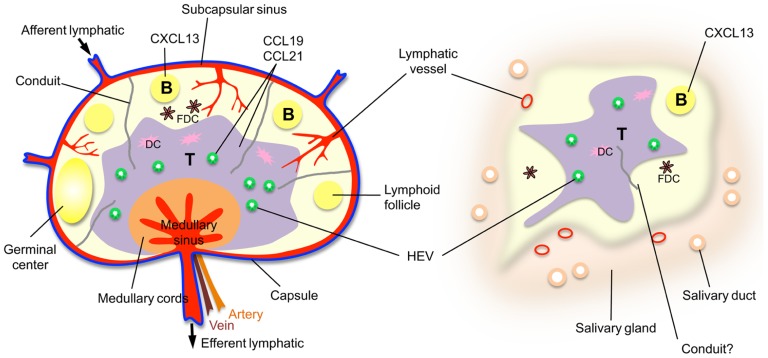
**A comparison of a lymph node with a TLO, a salivary gland from an individual with Sjögren’s syndrome.** Note that the HEVs and lymphatic vessels are colored to indicate the existence of mice with green fluorescent HEVs (Bentley et al., 2011) and red fluorescent lymphatic vessels (Truman et al., 2012) that have been developed to facilitate *in vivo* imaging.

While SLOs arise during development at key locations in the body, chronic immune activity in the adult can give rise to similar organized accumulations of lymphoid cells in almost any non-lymphoid tissue. These TLOs closely resemble SLOs (particularly LNs) with regard to cellular composition, organization, chemokines, and vasculature. TLOs have been noted during chronic inflammatory processes, including autoimmunity, chronic graft rejection, persistent infection (summarized in Drayton et al., 2006), atherosclerosis (Grabner et al., 2009), and cancer (Martinet et al., 2011; Bergomas et al., 2012). TLOs can also be induced experimentally by tissue-specific expression of certain inflammatory mediators (summarized in Drayton et al., 2006), including members of the lymphotoxin (LT) family (Kratz et al., 1996; Drayton et al., 2003), cytokines crucial for lymphoid organ development and maintenance (Ruddle and Akirav, 2009). Like SLOs, TLOs display organized leukocyte subtype compartmentalization driven by lymphoid chemokines (CCL19, CCL21, and CXCL13), the formation of germinal centers, and a highly organized vascular system, including HEVs, LVs, and perhaps conduits (**Figure 1**). Data suggesting that TLOs function as local sites of antigen presentation and lymphocyte activation, including somatic hypermutation and class switching in B cells (Schroder et al., 1996), suggest that they facilitate local anti-microbial responses, as well as epitope spreading (McMahon et al., 2005) and autoimmune exacerbation.

Not all inflammatory infiltrates organize into TLOs. Furthermore, in some instance, TLOs can progress from a relatively benign to a destructive phase. In type 1 diabetes in the NOD mouse, initial pancreatic infiltrates are characterized by HEV development and minimal islet destruction, whereas later stages demonstrate frank islet destruction and diabetes (Andre et al., 1996). In this model, pancreatic TLOs disappear after removal of the β cell antigen, being replaced by tissue fibroblasts.

In the following sections, we present a detailed comparison of the stromal characteristics of SLOs and TLOs.

## LYMPHOID TISSUE ORGANIZER CELLS

In the early stages of SLO development, various initiating events can activate stromal cells, called lymphoid tissue organizer (LTo) cells (CD4^–^CD3^–^IL7^+^VCAM-1^+^CXCL13^+^LTβR^+^), which recent data suggest may be derived from adipocyte precursors (Bénézech et al., 2012). LTo cells then activate lymphoid tissue inducer (LTi) cells (CD4^+^CD3^–^LTαβ^+^, IL7R^+^ CXCR5^+^) and they in turn, by their cytokine production, activate LTo cells. Although, LTo cells have not yet been isolated from TLOs, LTβR has been shown to induce aortic smooth muscle cells, which are implicated in TLOs in atherosclerosis (Grabner et al., 2009), to express some genes characteristic of LTos, including CXCL13 and VCAM-1. Both CXCL13 (Hjelmstrom et al., 2000) and VCAM-1 (Kratz et al., 1996) have been observed in TLOs. However, it is possible that signals from other cells could take over the function of LTos in adult TLOs and LNs. Cells with LTi characteristics have been noted in NOD (Evans and Kim, 2009) and RIPCXCL13 (Link et al., 2011) pancreatic TLOs suggesting that they may play a role in the development of these ectopic tissues.

## FOLLICULAR DENDRITIC CELLS

Follicular DCs form a network supporting B cell follicles in LNs. They are characterized by expression of complement receptors (CR1 and CR2), FcγRIIb, and markers defined by the antibodies FDCM1, FDCM2, and C4. Their roles include capture of antigen–antibody complexes for presentation to B cells and expression of the chemokine, CXCL13, which draws B cells and T follicular helper cells to the follicles via CXCR5.

Follicular DCs (FDCM1^+^CR1^+^) have been noted in TLOs. Their reticular network and association with B cells (Drayton et al., 2003) in TLOs suggest functional similarities with SLOs. As noted above, CXCL13 is found in TLOs, including those associated with *Helicobacter pylori* (Mazzucchelli et al., 1999), rheumatoid arthritis (Loetscher and Moser, 2002), Sjögren’s syndrome (Barone et al., 2005), inflammatory transgenes (Kratz et al., 1996; Hjelmstrom et al., 2000; Drayton et al., 2003), and cancer (Bergomas et al., 2012). However, although FDCs are a major source of CXCL13 in LNs, monocytes and macrophages have also been shown to secrete this chemokine in the TLOs associated with rheumatoid arthritis and ulcerative colitis (Carlsen et al., 2004), suggesting additional chemokine sources in chronic inflammation, including LTo cells as noted above. Antigen presentation by FDCs has not been investigated in TLOs.

## FIBROBLASTIC RETICULAR CELLS AND CONDUITS

The T cell zone of SLOs contains a dense, three-dimensional network of stromal cells called FRCs. This subset of LN stromal cells expresses podoplanin (gp38) but not the lymphatic and blood endothelial cell marker, PECAM (CD31; Turley et al., 2010). FRCs produce the extracellular matrix scaffolding that forms a series of very fine microvessels called conduits, detected using an antibody (ER-TR7) that recognizes an undefined antigen (Katakai et al., 2004). Conduits extend from the subcapsular sinus through the LN cortex, with greatest density in the paracortical T cell zone, and terminate at HEVs. These microchannels are approximately 4–5 nm in diameter and contain a dense network of collagen fibers that collectively limit access to molecules over 70 kDa in size (Sixt et al., 2005).

In addition to their role in forming conduits, FRCs also participate in the establishment and organization of lymphoid organ microenvironments (reviewed in Turley et al., 2010). They are the primary source of the T cell zone-restricted chemokines CCL19 and CCL21, both ligands for CCR7, present on T cells and DCs. These chemokines establish the T zone boundary, facilitate T cell and DC recruitment, and enhance DC maturation and function. FRCs also produce IL-7, critical for naïve T cell survival. The FRC network forms the three-dimensional scaffold along which T cells and DCs migrate; via their shared association with FRCs, interactions between naïve T cells and DCs expressing cognate antigen are facilitated. FRCs express transcripts for some self-antigens and it is has been suggested that they may be involved in self-tolerance (Lee et al., 2007). Additional LN stromal cells, termed eTACs, which are gp38^–^ERTR-7^–^, have also been reported to present self-antigens (Gardner et al., 2008).

Conduits (Anderson and Anderson, 1975) are believed to serve three key roles in SLOs (reviewed in Mueller and Ahmed, 2008). Given their narrow diameter and positioning, conduits can funnel small lymph-borne antigens from afferent lymphatics to the T cell zone, where FRC-associated resident DCs can receive them. Movement through these microvessels is much more rapid than that which could be achieved by simple filtration through the cell-dense tissue of the LN. Although this reticular network is less dense in the B cell zone, conduits may also serve as a pathway for low molecular weight antigens to reach follicles and facilitate B cell responses (Roozendaal et al., 2009). Finally, conduits serve as channels for transmitting small molecule chemical signals, such as chemoattractants from the surrounding tissue, to the paracortical region of the LN, ultimately reaching the endothelial cells lining HEVs.

**FIGURE 2:**
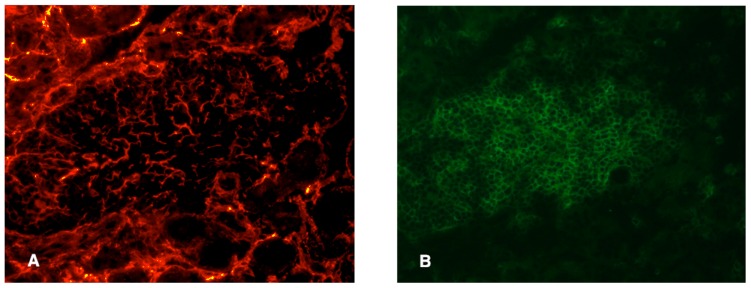
**Presumptive conduit in a RIPLTα TLO**. Infiltrate of a RIPLTα mouse with a kidney infiltrate, stained with ER-TR7 (red; **A**) anti-CD3 (green; **B**). Note the presence of the fine network of ER-TR7^+^ cells underlying the infiltrate.

Fibroblastic reticular cells and conduit-like structures have been noted in several studies of TLOs. Link et al. (2011) visualized these stromal elements using two-photon microscopy to detect injected low molecular weight fluorescent dyes, as well as via confocal microscopy and staining with ER-TR7 antibody. In TLOs from NOD or rat insulin promoter (RIP) LTα and RIPCXCL13 transgenic mice FRC-like stromal cells were observed, with the greatest density of staining in the T cell enriched areas of the TLO. In another study of NOD mice, ER-TR7 reactivity was found surrounding pancreatic islets and throughout the acinar tissue, consistent with the expression of this marker on fibroblasts. However, it was also found within the infiltrate (Penaranda et al., 2010), suggesting a supporting stromal network does exist in this case as well. Likewise, using fluorescently labeled ER-TR7 antibodies, we have observed dense staining patterns that resemble conduits in the T cell rich areas of the kidney infiltrates of RIPLTα mice (**Figure 2**). However, similar, although less dense, staining could also be seen in kidney samples from control mice, consistent with this as a fibroblast marker. Pulmonary arteries of patients with idiopathic pulmonary arterial hypertension who develop circulating autoantibodies to various vascular self-antigen include TLO-like structures and ER-TR7 antibody positivity (Perros et al., 2012). In non-lymphoid human tissues undergoing chronic inflammation (e.g., the liver of patients with primary biliary cirrhosis or the salivary glands of Sjögren’s patients), conduit-like gp38^+^CD31^–^LYVE-1^–^ networks similar to those in LNs were seen in most of the T cell dense regions, but not in control tissues.

Although cells with the characteristics of FRCs have been observed in TLOs, their functions are unclear. In the absence of a defining capsule and subcapsular sinus it is more difficult to appreciate these structures as conduits transporting low molecular molecules. Whether they can secrete tissue-organizing cytokines, serve as scaffolding for the migration of T and B cells or channel low molecular weigh substances toward TLO compartments remain unanswered questions. Cells phenotypically similar to eTACs have been noted in NOD pancreatic TLOs (Gardner et al., 2008) raising the question as to whether self-antigen presentation by stromal cells occurs in TLOs and exacerbates or induces tolerance in those locations.

## LYMPHATIC VESSELS

Afferent LVs deliver antigen and APCs to LNs and efferent vessels serve as routes for cell traffic to downstream LNs. Recently, an additional LV function was described: expression of self-antigen under the control of AIRE for presentation to T cells (Cohen et al., 2010). In LNs, DCs accumulate in the subcapsular sinus and transmigrate through the floor of that sinus into the T cell zone, while T cells access the parenchyma of the LN through the peripheral medullary sinuses (Braun et al., 2011). Egress from the LN is regulated in part by the high concentration of S_1_P in the lymph and the re-expression of the receptor, S_1_P_1_, on T cells as they leave the node and enter into the efferent lymph (Cyster and Schwab, 2012).

The presence of vessels with typical lymphatic markers, including LYVE-1 and Prox1, has been documented in the TLOs arising in both clinical settings (autoimmune disease and chronic graft rejection) and experimental models (summarized in Kerjaschki et al., 2004; Drayton et al., 2006; Furtado et al., 2007; Liao et al., 2007; Grabner et al., 2009; Mounzer et al., 2010). Although some macrophages also express LYVE-1, additional characteristics including gp38 expression, thin walls, and absence of red blood cells convincingly demonstrate their identity as LVs.

It has been suggested that TLOs differ from SLOs by the absence of a capsule. Thus, the trafficking patterns analogous to those in LNs (Braun et al., 2011), with the DCs percolating and T cells migrating through the peripheral medullary sinus to the parenchyma, might not occur in TLOs. However, TLOs in a variety of chronic kidney diseases (Mandache and Penescu, 2011) are in contact with a fibrous capsule, suggesting that, similar modes of migration in TLOs may be possible.

During ontogeny, LVs develop after the embryonic blood vessels have formed, sprouting off from the cardinal vein (Srinivasan et al., 2007), orchestrated by the homeobox genes (Sox18 and Prox1) and growth factors and their receptors (VEGFC and VEGFR3), and requiring platelets (Abtahian et al., 2003). LTα (Mounzer et al., 2010), DCs and T and B cells have been implicated in the regulation of LVs in inflammation (Angeli et al., 2006; Liao and Ruddle, 2006; Chyou et al., 2011). Regulation of lymphangiogenesis in TLOs is less well understood, although DCs have been implicated in a transgenic model of thyroiditis (Muniz et al., 2011). LTα regulates LVs in RIPLTαTLOs with less dependence on the LTαβ complex (Mounzer et al., 2010). On the other hand in the CXCL13 induced model of thyroiditis, LVs are inhibited by treatment with a LTβR-Ig (Furtado et al., 2007).

In some TLOs LVs are packed with lymphocytes (Liao et al., 2007) suggesting that they may act to transport activated lymphocytes to downstream LNs, similar to their function in SLOs. Continual administration of FTY720, an S_1_P receptor agonist that prevents egress of lymphocytes from LNs (Mandala et al., 2002), prevents diabetes in NOD mice (Penaranda et al., 2010). This is only effective if the mice have already developed pancreatic TLOs. Treatment results in additional accumulation of lymphocytes in the pancreatic TLOs, which is reversed upon cessation, resulting in rapid islet destruction and diabetes. These data suggest that lymphocyte trafficking through LVs in TLOs in NOD mice is under regulation of the lymph S_1_P gradient and expression of its receptor by T cells as in a canonical LN. The recent advances in *in vivo* imaging of LNs with the development of mice with red fluorescent LVs (Truman et al., 2012) may allow further analysis of the function of LVs in TLOs.

## HIGH ENDOTHELIAL VENULES

High endothelial venules, which are post-capillary venules with cuboidal endothelium, are the sites of entry of naïve lymphocytes from the blood stream into LNs. This is accomplished by the interaction of molecules on the surface of lymphocytes with ligands expressed by HEVs. Peripheral node addressin (PNAd), defined by the MECA-79 antibody, is composed of any of a variety of chemically modified core glycoproteins, including GlyCAM-1, CD34, Sgp200, and podocalyxin, to become functional L-selectin ligands (Rosen, 2004). The several enzymes that mediate these post-translational modifications include FucT-IV, FucT-VII, and GlcNAc6ST2 (also called HEC-6ST, LSST, GST-3, HEC-GlcNAc6ST, gene name *Chst4*; Hiraoka et al., 1999, 2004; Hemmerich et al., 2001a,b; Homeister et al., 2001), GlcNAc6ST2 is uniquely expressed in high endothelial cells, with the exception of a population of cells in the intestine (Liao et al., 2007; Kawashima et al., 2009). Binding to PNAd slows down (tethers) L-selectin^hi^ lymphocytes in their progress through the blood vessels, allowing interaction with chemokines and integrins, eventually facilitating migration of lymphocytes toward chemokines located in the paracortical region (T cells, DCs) or cortex (B cells). PNAd rapidly replaces MAdCAM-1 after birth in mouse peripheral LNs (Mebius et al., 1997), but is expressed in mucosal LNs together with MAdCAM-1, the ligand for the integrin α_4_β_7_.

High endothelial venules are prominent features of TLOs and their presence can be considered the defining characteristic that distinguishes these organized structures from other forms of inflammatory infiltrate, as they are so crucial for the entrance of naïve cells. HEVs in TLOs (summarized in Drayton et al., 2006) are characterized by expression of MAdCAM-1, PNAd, and chemokines, particularly CCL21 (Hjelmstrom et al., 2000), as well as the crucial sulfating enzyme GlcNAc6ST2 (Bistrup et al., 2004). HEVs have also been noted in the inflammatory infiltrates associated with atherosclerosis (Grabner et al., 2009) and several tumors (Martinet et al., 2011; Bergomas et al., 2012). In fact, the presence of high numbers of these vessels is associated with improved clinical outcomes in breast cancer (longer disease free survival and reduced metastases; Martinet et al., 2011), suggesting that the HEVs in tumor TLOs could enhance the entrance and priming of naïve lymphocytes into effector cells at the tumor site and lead to improved outcome.

Regulation of HEVs in TLOs appears to be similar to that in SLOs. In LNs, LTα_3_ regulates MAdCAM-1 (Cuff et al., 1998) and the LTα_1_β_2_ complex regulates PNAd (Drayton et al., 2003, 2004; Browning et al., 2005) through the alternative NFκB pathway (Drayton et al., 2004). An LTβ antagonist, LTβR-Ig, inhibits HEVs in LNs (Browning et al., 2005; Liao and Ruddle, 2006; Liao et al., 2007) and also in the TLOs that arise in salivary and lacrimal glands of NOD mice (Fava et al., 2011a,b).

High endothelial venules function in LNs as entry sites for naïve cells. A similar portal function is assumed in TLOs because of the presence of naïve cells at those sites (Kratz et al., 1996) and evidence of epitope spreading (Miller et al., 2007). However, the actual migration of naïve lymphocytes from the blood stream via HEVs into the parenchyma has not yet been visualized in real time in TLOs. Our development of mice with green fluorescent HEVs (Bentley et al., 2011) and their *in vivo* imaging in LNs (Truman et al., 2012) will enable similar analysis in TLOs and resolve whether HEVs function as entry points for naïve cells.

## IMPLICATIONS

A better understanding of the stromal structure in TLOs could lead the way to therapeutics directed specifically at such structures. Likewise, a clearer appreciation of the inflammatory signals that drive the organization of stromal cells in non-lymphoid tissues into TLO-like structures might allow the design of therapeutic interventions to impede the progression of chronic inflammation, including autoimmune activity. Local interference with the chemokine signals that recruit and organize T and B cells into functional pro-inflammatory structures in non-lymphoid tissues might dampen autoimmune responses and allow for more effective conventional therapy for autoimmune diseases. Since regulatory T cells have been observed in some TLOs, a better understanding of and ability to enhance the tolerance inducing capabilities of APCs might help restrain self-reactivity, limiting exacerbation of autoimmune disease. Treatment with agents that enhance the alternative NF-κB pathway could encourage the development of HEVs in tumors, enhancing the entrance of naïve cells and providing useful new strategies in cancer. Although this concept is actually in contradistinction to treatments currently used to prevent angiogenesis in the tumor setting, understanding the complexity of the stroma in TLOs could provide creative new approaches to disease control.

## Conflict of Interest Statement

The authors declare that the research was conducted in the absence of any commercial or financial relationships that could be construed as a potential conflict of interest
